# Supplementary material for: The apiosyltransferase celery UGT94AX1 catalyzes the biosynthesis of the flavone glycoside apiin
Source: Plant Physiol. 2023 Jul 11;193(3):1758–71. doi: 10.1093/plphys/kiad402 (PMC10602602; doi:10.1093/plphys/kiad402)
Supplement: kiad402_Supplementary_Data [file kiad402_supplementary_data.pdf]

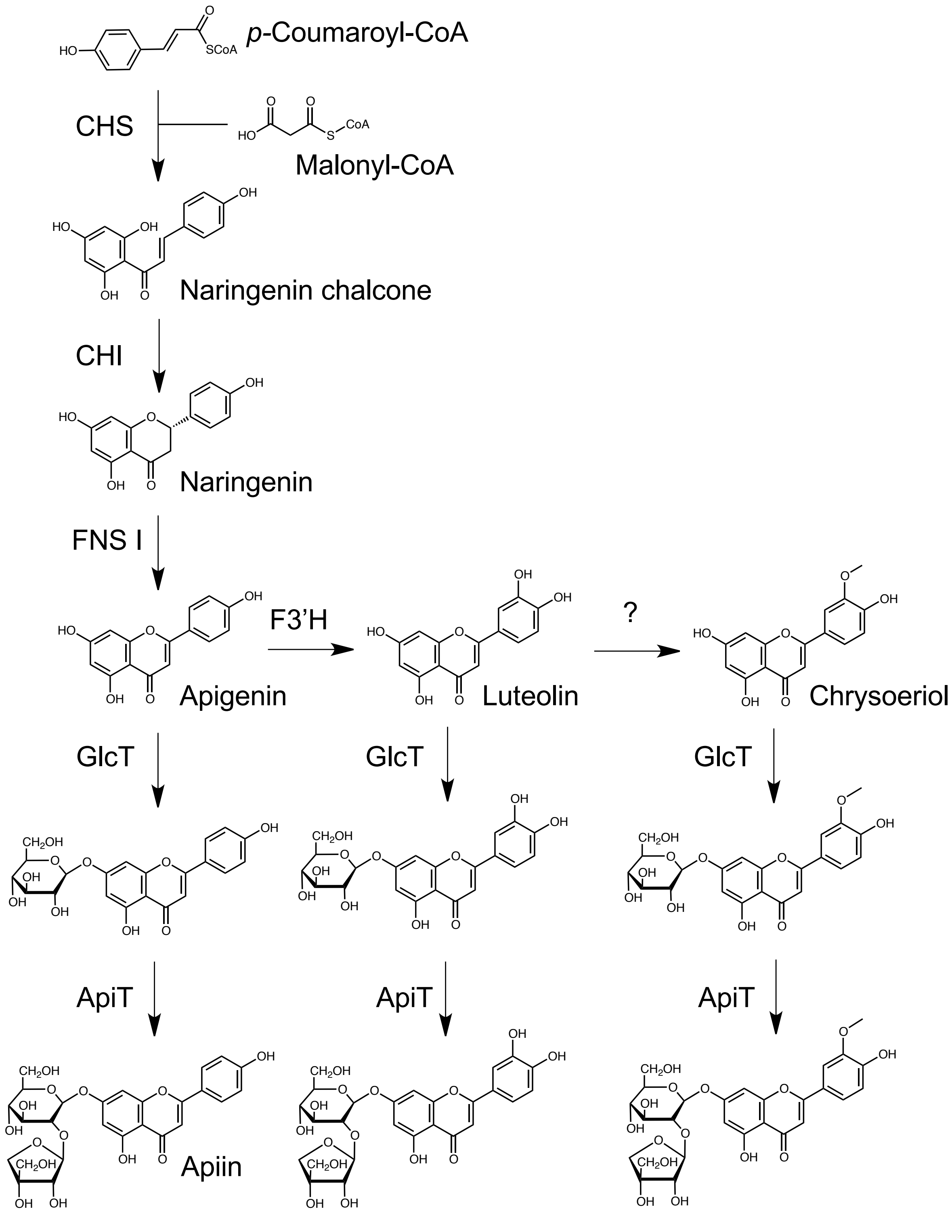

Supplemental Figure S1. Biosynthesis of apiosylated flavones in celery. CHS, chalcone synthase; CHI, chalcone isomerase; FNSI, flavone synthase I; F3′H, flavonoid 3′ hydroxylase; GlcT, glucosyltransferase; ApiT, apiosyltransferase.

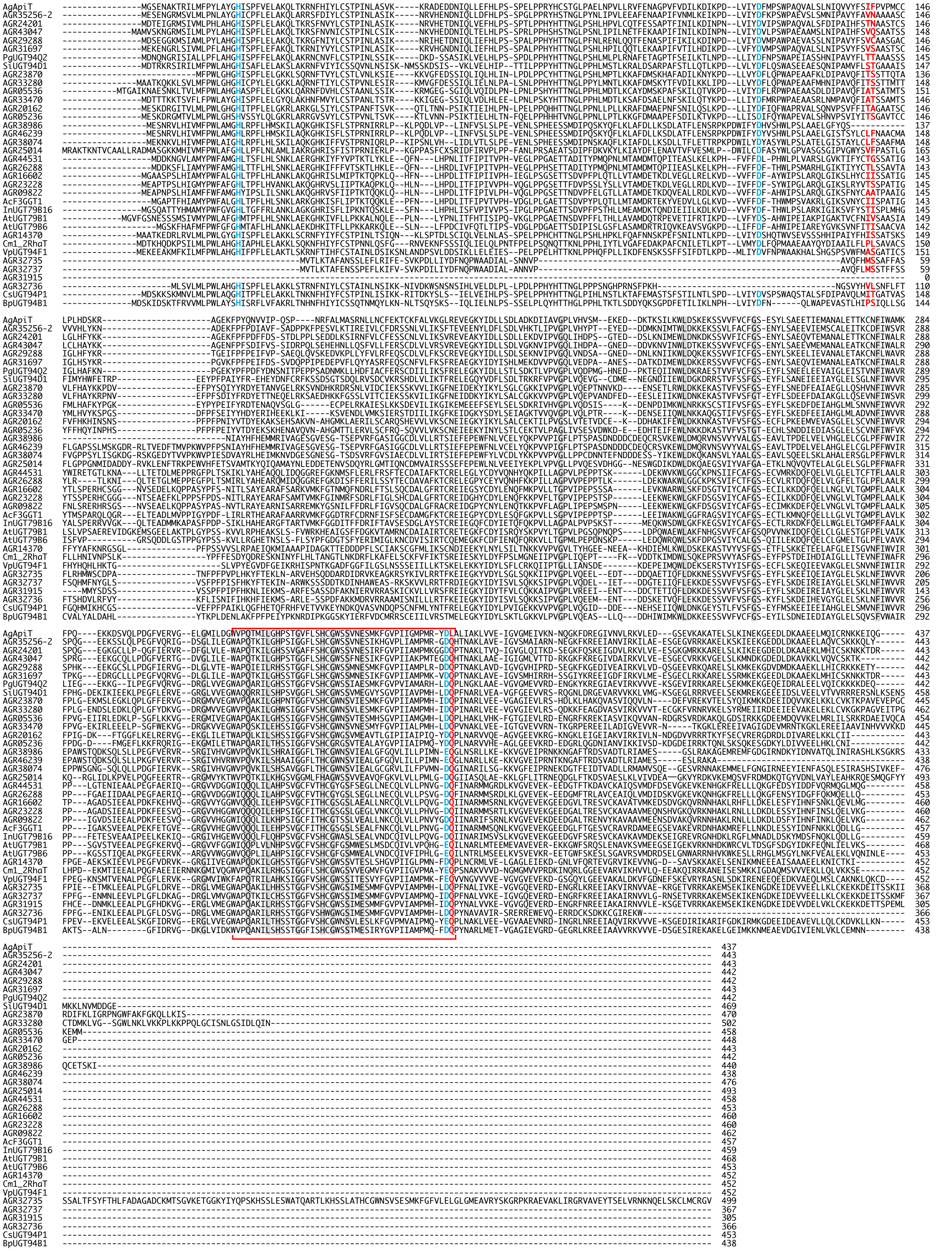

Supplemental Figure S2. Amino acid sequence alignment of AgApiT, celery GGTs, and representative plant GGTs that were biochemically characterized. Sequences were aligned with ClustalW. The amino acid residues involved in the recognition of apiose residue for AgApiT (Ile139, Phe140, and Leu356) and their corresponding residues in each enzyme are shown in red. The putative catalytic amino acid residues are shown in blue. The conserved amino acid residues are shaded. The PGPS box conserved in GGTs is marked with a red bar.

Supplemental Table S1. Nucleotide sequences of oligonucleotides used in this study.

Primer name	Nucleotide sequence (5′-3′)
AgApiTpCold_F	CCATCCATATGGGATCGGAAAACGCGAAA
AgApiTpCold_R	CCATCTCTAGATTAGCCCTGGATTTCCTT
Agr35256-2pCold_F	GAAGGTAGGCATATGGAAAGCGAAAATGGGCGT
Agr35256-2pCold_R	CAGCTATATTAACTCGAGGGATCCGAATTCAAGCTTGTC
AgApiT-I139S_F	GTCTACTTCAGCTCCTTTCCAGTCCCAATG
AgApiT-I139S_R	CATTGGGACTGGAAAGGAGCTGAAGTAGAC
AgApiT-I139T_F	GTCTACTTCAGCACCTTTCCAGTCCCAATG
AgApiT-I139T_R	CATTGGGACTGGAAAGGTGCTGAAGTAGAC
AgApiT-I139V_F	GTCTACTTCAGCGTCTTTCCAGTCCCAATG
AgApiT-I139V_R	CATTGGGACTGGAAAGACGCTGAAGTAGAC
AgApiT-F140T_F	AGCATCACACCAGTCCCAATGTGCTGT
AgApiT-F140T_R	GACTGGTGTGATGCTGAAGTAGACGAC
AgApiT-F140I_F	AGCATCATTCCAGTCCCAATGTGCTGT
AgApiT-F140I_R	GACTGGAATGATGCTGAAGTAGACGAC
AgApiT-F140V_F	AGCATCGTTCCAGTCCCAATGTGCTGT
AgApiT-F140V_R	GACTGGAACGATGCTGAAGTAGACGAC
AgApiT-L356I_F	CATGCGTTACGATATAGCCTTGATTGCGAA
AgApiT-L356I_R	TTCGCAATCAAGGCTATATCGTAACGCATG
AgApiT-L356Q_F	ATGCCCATGCGTTACGATCAGGCCTTG
AgApiT-L356Q_R	CGCAATCAAGGCCTGATCGTAACGCAT
AgApiT-L356T_F	CATGCGTTACGATACCGCCTTGATTGC
AgApiT-L356T_R	AATCAAGGCGGTATCGTAACGCATGGG
AgApiT_F	TCGGTGGTGTTTGTGTGTTTTGG
AgApiT_R	ACTCCTCAGCAGCCTTATCGAGA
FNS I_F	TGGTGAAAAGAGAGGCGGCTTTA
FNS I_R	CCGGGTTCTGGAAAGTTGCAATT
GAPDH_F	CTAGCAACTTTGGCCTCCAG
GAPDH_R	GCGATCCAATTCCACTGTCT

Mutated codons are underlined.
